# Wound healing after excision of subcutaneous tumors treated with near‐infrared photoimmunotherapy

**DOI:** 10.1002/cam4.3247

**Published:** 2020-06-24

**Authors:** Adrian Rosenberg, Fuyuki Inagaki, Takuya Kato, Ryuhei Okada, Hiroaki Wakiyama, Aki Furusawa, Peter L. Choyke, Hisataka Kobayashi

**Affiliations:** ^1^ Molecular Imaging Program Center for Cancer Research National Cancer Institute National Institutes of Health Bethesda MD USA

**Keywords:** near‐infrared photoimmunotherapy, neoadjuvant, reactive oxygen species, surgery, wound healing

## Abstract

Near‐infrared photoimmunotherapy (NIR‐PIT) is a novel cancer therapy that employs a combination of infrared light and tumor‐targeted monoclonal antibody‐photoabsorber conjugates to cause both direct tumor necrosis and immunogenic cell death. NIR‐PIT may have potential in the perioperative setting before surgery, and therefore it is important to know the effect of NIR‐PIT on wound healing. Fifty mice were implanted with subcutaneous xenografts of N87 human gastric cancer cells, and tumors were excised after reaching a predetermined size. After excision, 30 mice were split into three groups: Controls, NIR‐PIT 1 day prior to surgery and NIR‐PIT 3 days prior to surgery. The quantity of reactive oxygen species (ROS) in each wound was measured on Postoperative Days 2 and 4, and mice were monitored weekly for 4 weeks for evidence of local tumor recurrence as well as clinical evidence of wound healing complications (eg, dehiscence, infection). The remaining 20 mice (10 controls, 10 treated with NIR‐PIT 1 day prior to surgery) were sacrificed on either Postoperative Day 7 or 14, the skin around wounds were excised, and tensile strength was measured with a digital force gauge. There were no significant differences between treatment and control groups with respect to wound ROS levels, wound tensile strength, local tumor recurrence, or postoperative complication rates (*P* > .05). In conclusion, neoadjuvant (pre‐operative) NIR‐PIT shows no evidence of adverse wound healing effects, and it is likely a safe adjunctive treatment to surgery. Postoperative use of NIR‐PIT merits investigation.

## Graphical abstract

Near‐infrared photoimmunotherapy (NIR‐PIT), which is now under world‐wide phase 3 trial including in Japan, may be useful in the perioperative setting either before or after tumor excision that should be rational because NIR‐PIT could minimize tumor volume and initiate host antitumor immunity for safe and successful outcome. In order to apply this neoadjuvant NIR‐PIT before the surgery, only the concern of surgeons is if wound healing is hampered by preoperative NIR‐PIT. Therefore, in this study, in order to respond high demands from many surgeons, the effect of NIR‐PIT on wound healing was measured using reactive oxygen species monitoring and wound tensile strength measurements and shown to have no difference between treatment and control mice.

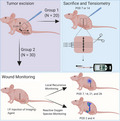

## 1 INTRODUCTION

Cancer continues to be a major global health challenge.^1^ Near‐infrared photoimmunotherapy (NIR‐PIT) is a novel cancer therapy that utilized antibody‐photoabsorber conjugate (APC) and near infrared light. NIR‐PIT causes cancer‐cell selective cytotoxicity with minimal off‐target effects and has successfully targeted a diversity of cancers in the preclinical setting.^2^, ^3^ NIR‐PIT triggers an intensely immunogenic cell death^4^ that leads to a rapid inflammatory response followed by a cancer antigen‐specific immune response. This latter response plays a central role in the treatment's overall efficacy.^5^, ^6^, ^7^ NIR‐PIT may have a particular role in the neoadjuvant or adjuvant settings of solid tumor surgery, including downstaging or debulking of tumors *prior* to surgery or as a method of removing residual or microscopic tumor(s) immediately *after* surgery from excision sites or large surfaces (eg, peritoneum).^8^, ^9^, ^10^ In either case, predicting the effect of NIR‐PIT on wound healing would be important.

Wound healing is a complex physiologic process that involves overlapping phases of inflammation, proliferation, and tissue remodeling.^11^ Wound healing is negatively affected by excessive inflammation in both animal models^12^, ^13^ and humans.^14^, ^15^ The immune activation stimulated by NIR‐PIT may therefore affect this physiologic process. One quantitative means of measuring this process includes bioluminescence imaging using the chemiluminescent Luminol derivative L‐012 (C_13_H_8_CIN_4_NaO_2_).^16^, ^17^ Luminol is a relatively new means of quantifying reactive oxygen species (ROS) in murine wound models,^18^ and differences in wound ROS with vs without NIR‐PIT would suggest a potential negative impact on wound healing. Direct measurement of wound tensile strength is another validated method of ascertaining interference in wound healing in murine models.^19^ Thus, using these methods, we investigated the effects of perioperative NIR‐PIT on postoperative wound healing in a murine model of cancer.

## 2 MATERIAL AND METHODS

### 2.1 Cell lines and culture

The HER2‐positive human gastric cancer cell line, N87GFP‐luc, was used for all studies. The cells express both Green Fluorescent Protein (GFP) and Luciferase (Luc). Cells were grown in RPMI 1640 (Life technologies) containing 10% fetal bovine serum (Life Technologies), 0.03% l‐glutamine, 100 units mL^−1^ penicillin and 100 mg mL^−1^ streptomycin in 5% CO_2_ at 37°C.

### 2.2 Reagents and APC synthesis

The monoclonal antibody (mAb) used for APC synthesis was Trastuzumab (Herceptin, Genentech), an IgG1 kappa, humanized mAb against HER2, maintained at 4°C in stock solution. The dye was IRDye 700Dx ester (IR700; C_74_H_96_N_12_Na_4_O_27_S_6_Si_3_, MW: 1954.22), and it was purchased from LI‐COR Bioscience. All other chemicals were of reagent grade.

One milligram (6.8 nmol) of Trastuzumab was incubated with 66.8 µg (34.2 nmol) IR700 (5 mmol L^−1^ in DMSO) in 0.1 mol L^−1^ Na_2_HPO_4_ (pH 8.6) at room temperature for 1 hour. The mixture was subsequently purified with a Sephadex G50 column (PD‐10; GE Healthcare). The protein concentrations were confirmed with Coomassie Plus Protein Assay Kit (Pierce Biotechnology) by measuring light absorption at 595 nm (8453 Value System; Agilent Technologies). The concentration of IR700 was measured by absorption with spectroscopy to confirm the average number of fluorophore molecules conjugated to each Trastuzumab molecule. APC solutions were individually diluted with PBS to achieve final concentrations of 500 µg mL^−1^.

### 2.3 NIR‐PIT on a mouse tumor model

**FIGURE 1:**
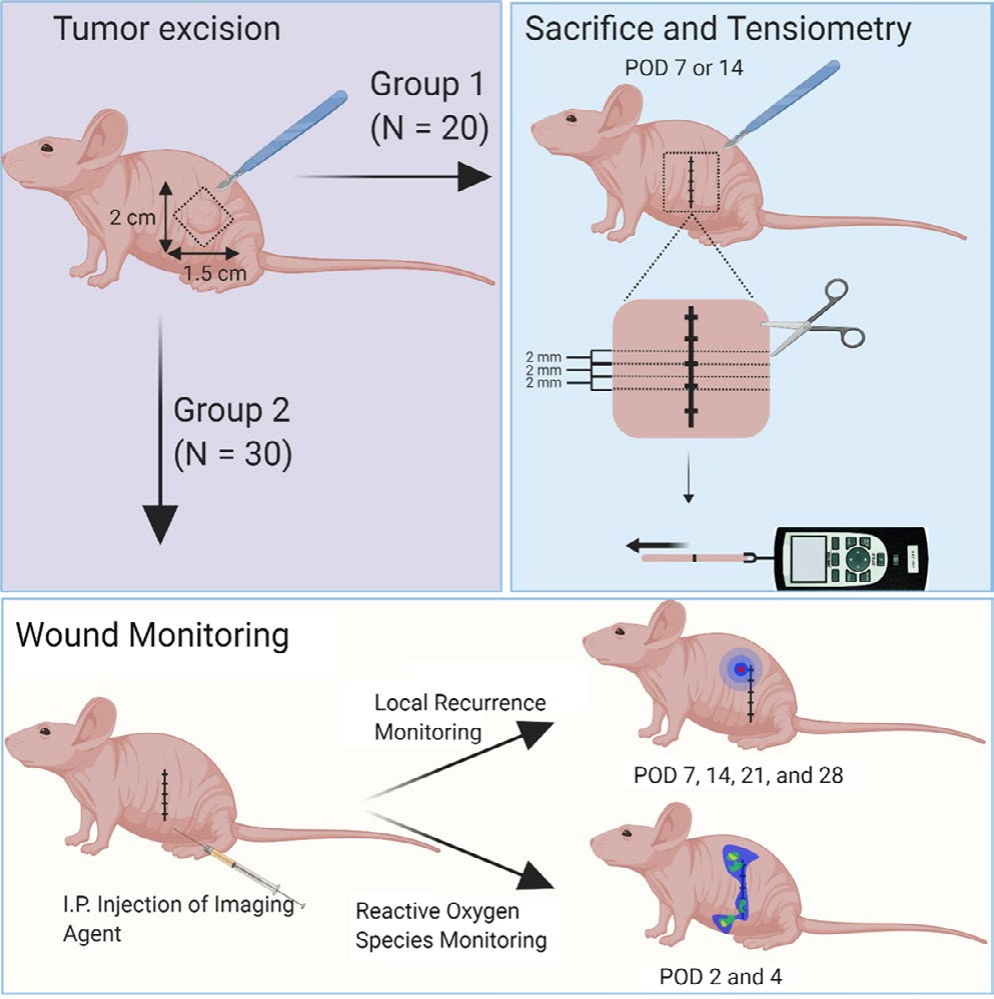
**Scheme for experimental planning**. POD, postoperative date

All in vivo procedures were conducted in compliance with the Guide for the Care and Use of Laboratory Animal Resources (1996), US National Research Council, and approved by the Institutional Animal Care and Use Committee. Female homozygote athymic nude mice aged 6‐8 weeks were purchased from Charles River (National Cancer Institute). During treatment, mice were anesthetized with isoflurane. 3 × 10^6^ N87GFP‐luc cells were injected subcutaneously in the left flank. In treatment mice, 10‐14 days after injection, mice with long‐axis tumor diameters between 5 and 9 mm and total tumor volume (as given by the formula 0.5 × length × width × depth) between 50 and 150 mm^3^ were injected with 100 µg APC through tail vein injection. Twenty‐four hours later, NIR laser light (690 ± 5 nm) was administered at a dose of 50 J cm^−2^ (Modulight Inc ML7710; Cylindrical Light Diffuser Model: R030). Experimental design after treatment is illustrated in Figure 1. Briefly, 20 mice were used for tension experiments (see below), 10 treatment mice (NIR 1 day prior to surgery) were evaluated at 7 (n = 5) or 14 (n = 5) days after surgery, and control mice were divided in the same manner. Thirty mice were monitored for local recurrence, with 10 receiving NIR‐PIT 3 days before surgery, 10 1 day before surgery, and 10 controls.

### 2.4 Survival surgery

#### 2.4.1 Surgery

Tumors were surgically excised at either Day 1 or 3 post‐NIR‐PIT in the experimental group, or at the time the tumor reached the same size for controls. Five minutes before incision, mice were simultaneously administered three medications intraperitoneally: 5 mg kg^−1^ of Xylazine and 2 mg kg^−1^ of Ketamine for intraoperative sedation and anesthesia, and 0.25 mg kg^−1^ Buprenorphine for postoperative pain control. Immediately prior to incision, tumor size was recorded and the left trunk, from foreleg to hindleg, was scrubbed with three rounds of alternating Betadine solution (10% Povidone Iodine, Purdue Pharma) and reagent grade Isopropyl Alcohol (Sigma Aldrich). A 2 × 1.5 cm excisional square was then marked around the tumor, and the skin and underlying tumor were excised. Intraoperative in vivo fluorescence imaging was then performed with the Maestro In Vivo Fluorescence Imager (Cri, Woburn, MA, USA) using a 445‐490 nm band pass excitation filter with a long pass emission filter over 515 nm to confirm complete tumor excision. Topical Bupivicaine drops were added to the wound up to a maximum of 8 mg kg^−1^ for intraoperative pain control if there was any evidence of pain (eg, twitching or increased respiratory rate). The excised GFP‐positive tumor was included in the images as a positive control. Mice were excluded if complete excision could not be verified after three attempts (n = 1) or if there was peritoneal invasion (n = 4). Wounds were closed with three to four simple interrupted sutures using 4‐0 nylon sutures (UNIFY). Immediately after wound closure, 4 mg kg^−1^ Ketoprofen was subcutaneously injected for further control of postoperative pain. Surgical instruments were autoclaved in 20‐minute cycles (AMSCO Century, Steris) at the beginning of each surgery day and were sterilized in between surgeries with a glass bead sterilizer (Germinator 500; Cellpoint Scientific).

#### 2.4.2 Postoperative care

Mice were monitored daily for pain in the first postoperative week and every 2 days thereafter. In the first intraoperative week, mice were housed individually and fitted with Elizabethan Collars (E‐Collars) (Kent Scientific Corporation) to prevent biting of sutures. To prevent ocular infections, E‐Collars were removed for 5 minutes under direct observation on the first and third days after surgery to allow for mouse self‐grooming. After the first week, E‐collars and any remaining sutures were removed. Mice were housed together after the second week.

### 2.5 Postoperative wound monitoring

#### 2.5.1 Reactive oxygen species monitoring

In the wound monitoring group (group 2), ROS monitoring was performed on both experimental and control mice on Postoperative Day 2 and 4. The bioluminescence of the ROS in each wound was imaged and quantified using a photon counting imaging system (PhotonIMAGER OPTIMA; Biospace Lab). The Luminol derivative L‐012 (FUJIFILM Wako Pure Chemical Corporation) was administered intraperitoneally (0.5 mg 20 g^−1^) 50 minutes prior to imaging. Imaging duration was 2 minutes. Regions of Interest (ROI) were placed over the wounds, and counts of Relative Light Units were analyzed using M3 Vision Software (Biospace Lab).

#### 2.5.2 Local recurrence monitoring and postoperative complication monitoring

In the wound monitoring group, mice were also monitored for local recurrence on Postoperative Days 7, 14, 21, and 28. Luciferin was intraperitoneally administered (15 mg mL^−1^, 200 µL) and bioluminescence (BLI) images were acquired 15 minutes later using a BLI camera (PhotonIMAGER Optima). Daily monitoring for postoperative complications revealed no instances of wound dehiscence or local or systemic infection.

#### 2.5.3 Wound tensile strength measurements

In Group 2, experimental and control mice were euthanized at either Postoperative Day 7 or Day 14, and a rectangle piece of skin encompassing the entire wound was excised and cut into multiple 2‐millimeter (mm)‐wide strips. The most peripheral strips (those from the dorsal and ventral wound ends) were not used. For each mouse, the tensile strength of three strips was measured by applying manual tension orthogonal to the direction of incision with opposite end of the strip clamped by a handheld digital force gauge (DST‐1A; IMADA Inc).

#### 2.5.4 Confirmatory imaging

Treatment groups of mice were imaged with IR700 fluorescence before and after laser therapy using an in vivo fluorescence imager (Pearl Imager, LI‐COR Bioscience) to confirm treatment efficacy. Complete resection of GFP positive tumors was confirmed with Maestro Imaging (see Section 2.4.1 above) and Local recurrence was monitored with a BLI camera (see Section 2.4.1, 2.5.2 above).

### 2.6 Statistical analysis

All statistical analyses were performed with GraphPad Prism (GraphPad Software). *P* values <.05 were considered significant.

## 3 RESULTS

### 3.1 Local recurrence rates and postoperative complications

There was no significant difference in recurrence rates, with a 10% local recurrence in each treatment group (n = 1 for each) and a 20% local recurrence rate in the control group (n = 2). There were no instances of thermal injury in the treatment groups, and no instances of wound infection or dehiscence in any mice (Table 1).

**TABLE 1:** Monitoring for local recurrence and postoperative complications

	Treatment: NIR 1 (N = 10)	Treatment: NIR 3 (N = 10)	Control (N = 10)
Local recurrence	10% (1)	10% (1)	20% (2)
Wound dehiscence	0 (0)	0 (0)	0 (0)
Wound infection	0 (0)	0 (0)	0 (0)
Thermal injury	0 (0)	0 (0)	Not applicable

### 3.2 Reactive oxygen species

**FIGURE 2:**
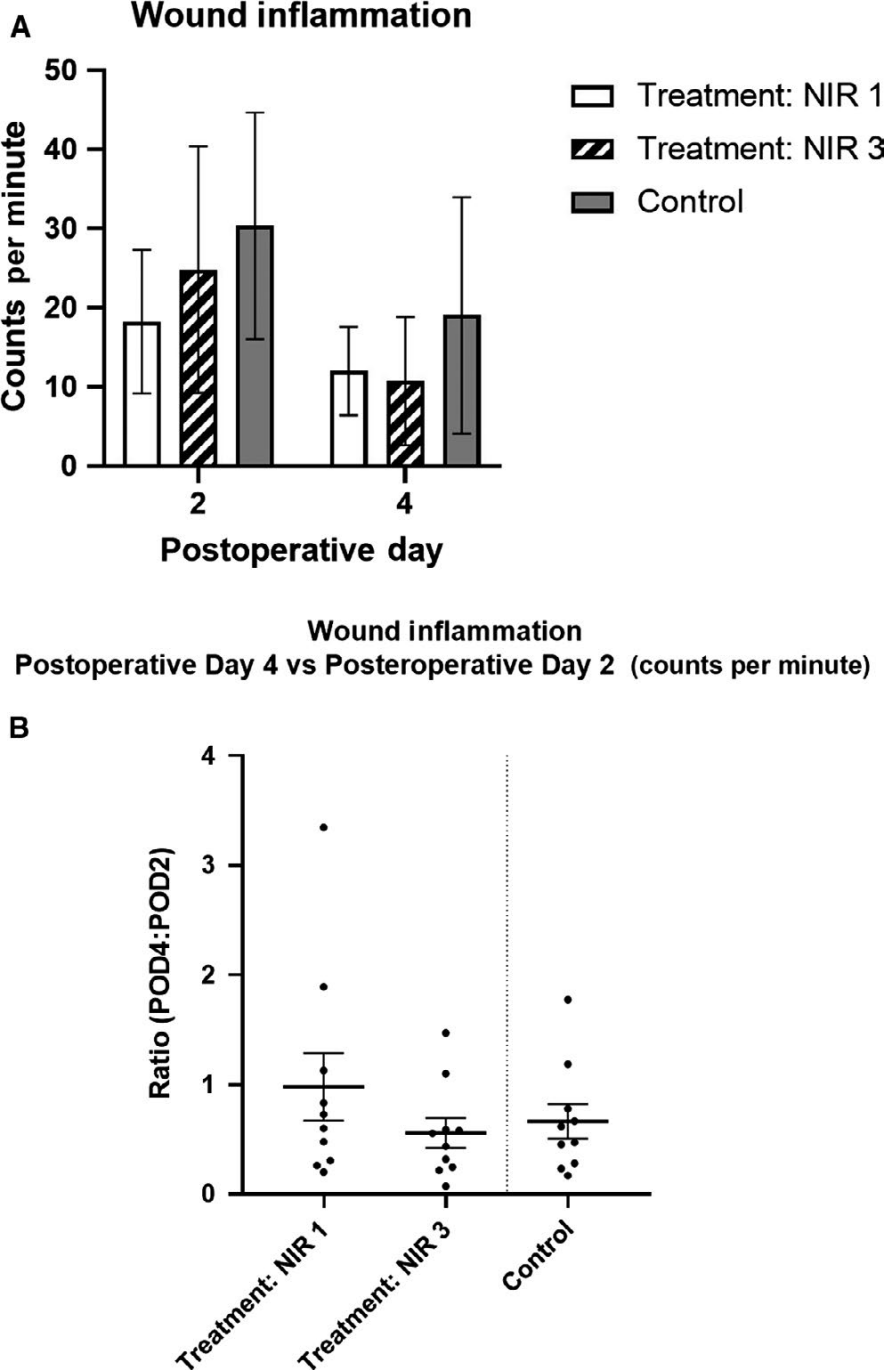
**Inflammation at the wound**. A, Mean wound inflammation was not significantly different between experimental groups (*P* > .05 for both POD 2 and 4; one‐way ANOVA). B, Ratio of POD4 to POD2 inflammation was not significantly different between treatment groups (*P* = .37; one‐way ANOVA). Error bars represent SEMs. Treatment NIR 1 and 3 = surgery performed day one and three post‐NIR‐PIT, respectively. Inflammation measured via photon imaging of reactive oxygen species 50 min after intraperitoneal administration of 0.5 g 20 g^−1^ dose of L‐012. POD, postoperative date

**FIGURE 3:**
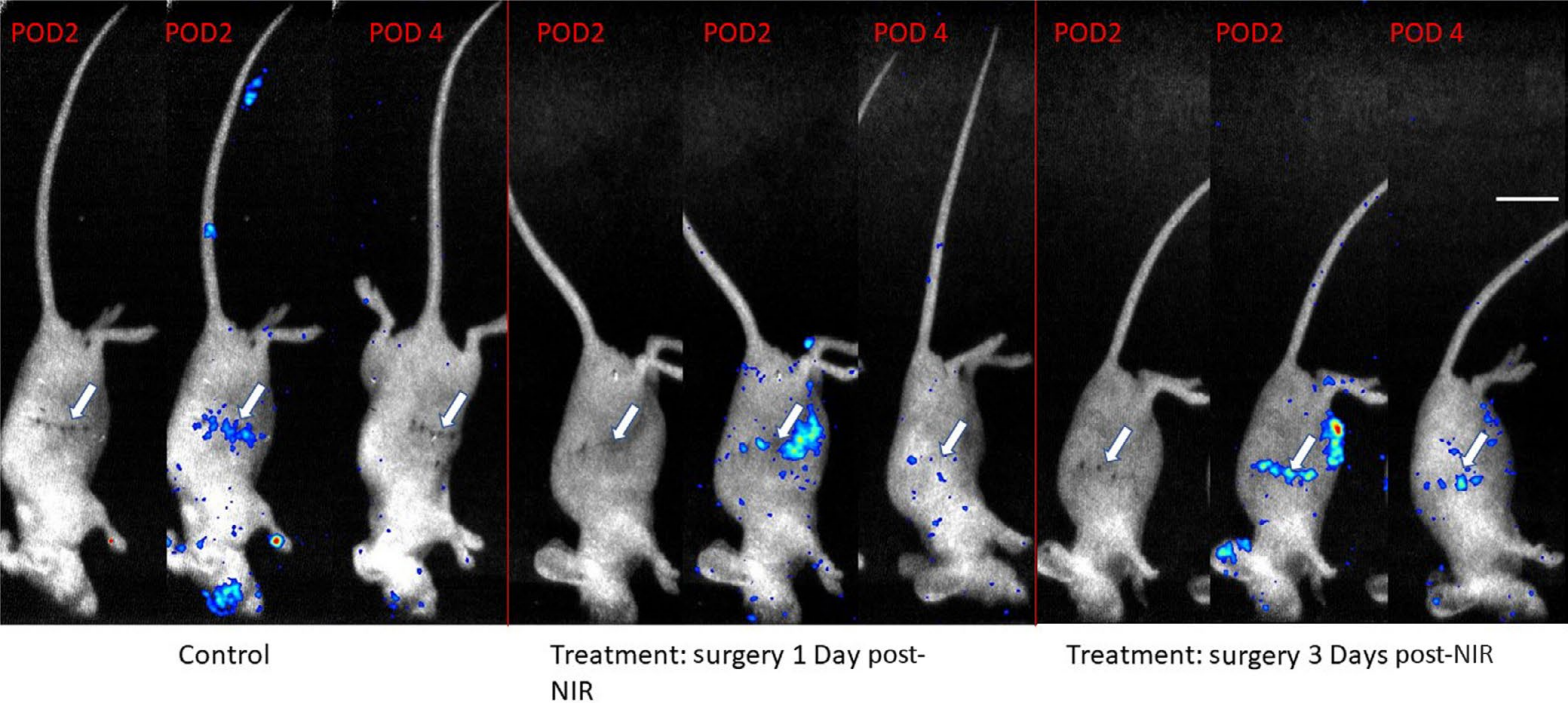
**Singlet oxygen production at the wound**. Representative mouse from each group, shown at Postoperative Day 2 (POD2) with white light images (to visualize the wound), Postoperative Day 2 with ROS signal included, and Postoperative Day 4 (POD4) with ROS signal included, respectively. Signal represents counts. Counts decrease significantly between POD 2 and POD 4. Reactive Oxygen Species elicited via intraperitoneal injection of L‐012 (0.5 mg 20 g^−1^) 50 min prior to imaging. Imaging window = 0.05‐0.50 × 10^−3^ counts for images in which ROS signal is included. In some mice, there is observable inflammation near the eyes (post‐operative inflammation secondary to Elizabethan collar usage, preventing self‐grooming), ears (location of hole‐punching for identification), and site of peritoneal injections. Arrows indicate where the tumors were located. Scale bar represents 1 cm

Mean photon counts in ROIs placed directly over the wounds were not significantly different between experimental groups (*P* > .05 for both postoperative date (POD) 2 and 4; one‐way analysis of variance (ANOVA), Figure 2A). Given that genetic variability among mice may cause variability in luminescence, the ratio of POD4 to POD2 photon counts was also measured. This ratio was not significantly different between treatment groups (*P* = .37; one‐way ANOVA, Figure 2B; representative images in Figure 3).

### 3.3 Wound tensile strength

**FIGURE 4:**
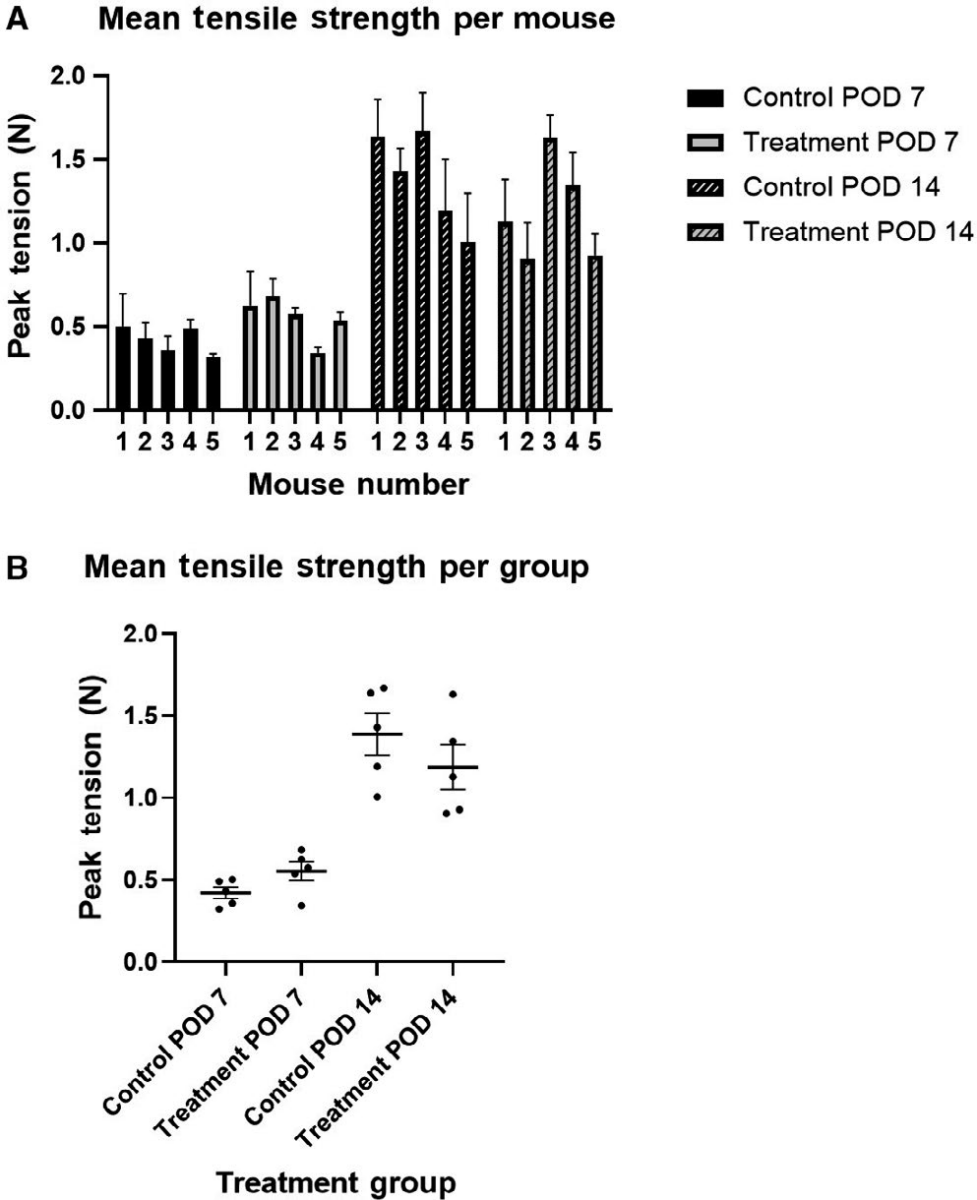
**Wound tensile strength**. A, Mean wound tension was not significantly different between mice in a given group (*P* > .05 for each group using one‐way ANOVA). B, Mean tension in control group vs treatment groups showed nonsignificant differences at their respective timepoints (mean tension POD 7: control = 0.42, treatment = 0.55, *P* = .096. POD 14: control = 1.39, Treatment = 1.19, *P* = .314; unpaired *t* test with Welch's correction). POD 7 and 14 = wound tension measured on Postoperative Days 7 and 14, respectively. N = 5 mice per group. N = 3 measurements per mouse, using three 2‐mm‐wide segments of skin tissue included wound. Tension applied orthogonal to direction of incision and suture. Error bars represent SEMs

Mean wound tension was not significantly different between mice in a given experimental group (*P* > .05 for each group using one‐way ANOVA, Figure 4A), demonstrating little intermouse variability in wound strength. Mean tension in the control group vs treatment groups showed nonsignificant differences at their respective timepoints (*P* > .05 for both Postoperative Day 7 and 14; unpaired *t* test with Welch's correction; Figure 4B).

### 3.4 Confirmatory imaging

**FIGURE 5:**
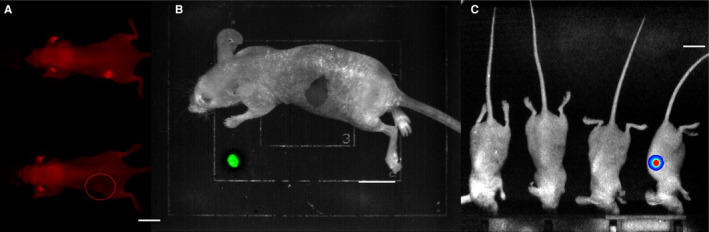
**Definition of imaging procedures**. A, Confirmation of antibody‐photoabsorber conjugate (APC) localization to the tumor and photochemical reaction to infrared light administration. Images taken immediately before (top) and after (lower) NIR light exposure, respectively. APC was administered 24 h prior to laser therapy. B, Intraoperative confirmation of complete tumor excision using in vivo fluorescence imaging of GFP‐positive tumors. Excised tumor included as positive control. C, Representative batch of control mice on Postoperative Day 7. Local recurrence detected in the rightmost mouse. Recurrence of Luciferase positive tumors is detected by intraperitoneal injection of Luciferin 15 min prior to imaging. Scale bars represent 1 cm

In vivo fluorescence imaging of IR700 before NIR light irradiation confirmed localization of APC in tumor. Missing IR700 fluorescence after NIR light irradiation confirmed successful photochemical reaction to NIR light (Figure 5A). GFP fluorescent imaging confirmed complete tumor resection (Figure 5B). Bioluminescence imaging showed local recurrence of luciferase expressing tumor (Figure 5C).

## 4 DISCUSSION

In this study, we show that in murine models of subcutaneously grafted tumors, NIR‐PIT either 1 or 3 days prior to excision did not affect the level of wound inflammation or strength of wounds after surgery. These results provide evidence that NIR‐PIT does not interfere with postsurgical healing and therefore, could be safely used in a perioperative clinical setting. An example of such use might be NIR‐PIT to de‐bulk tumors deemed too large for initial resection or reduce tumor volume in palliative treatments, without risk of adverse wound healing effects.

We used several measures of wound healing integrity to determine the effects of NIR‐PIT. A central component of wound monitoring was serial bioluminescence imaging to detect inflammation by ROS. In wound healing, basal levels of ROS are necessary to recruit lymphoid cells and promote angiogenesis and wound disinfection, though excessive ROS can cause oxidative stress at levels that inhibit cellular migration and fibroblast proliferation and promote cellular necrosis.^20^ Importantly, ROS are not simply surrogates of inflammation, but play a direct role in the repair and remodeling of wounds.^21^ In this study, ROS levels trended lower in the treatment group, but no significant difference between treatment and control groups on Postoperative Days 2 and 4 (*P* = .37) was observed.

Wound integrity was also measured with tensile strength measurement, which has a well‐established role for the evaluation of both human and animal wound healing.^22^, ^23^ The tensile strength data in this study provided important evidence of unimpaired wound healing. Wound tension at Postoperative Day 7 and 14 was identical between treatment and control mice, indicating that wounds were likely healing at similar rates between groups.

Inflammation at the wound site is also known to promote tumorigenesis and worsen recurrence rates.^24^, ^25^, ^26^ Wounds were therefore monitored for local recurrence over the course of 4 weeks using previously validated bioluminescence imaging techniques,^27^ and no significant difference in rates of recurrence were found for the treatment and control groups.

This study explored the use of NIR‐PIT in the preoperative setting, but postoperative use is another important question to be investigated. There is some evidence that infrared light using parameters similar to those of this study (685 ± 5 nm, 50 J cm^−2^) is safe and perhaps beneficial for wound healing. Pinheiro et al reported that a 40 J cm^−2^ dose of 685 nm light in excisional wound rat models showed histologic features of wound healing equal to that of controls, and in the 20 J cm^−2^ group they reported histologic features suggestive of improved wound healing.^28^ In incisional rat models, Suzuki and Takakuda also report increased tensile strength and increased collagen deposition 7 days after wounding when wounds were treated with 660 nm light 24 hours after surgery with doses as small as 1 and 5 J cm^−2^, though not at 10 J cm^−2^.^29^ Finally, Lackjavoca et al report improved wound healing with 670 nm 5 J cm^−2^ dosing of excisional wound rat models.^30^ Thus, the potential role of NIR‐PIT as a postsurgical adjuvant, with light administered even directly on fresh wounds, merits investigation.

## 5 LIMITATIONS AND CONSIDERATIONS

The differences between mouse and human skin are well documented.^31^ Additionally, although athymic nude mice were chosen because their skin is not confounded by the effects of hair growth on wound healing,^32^, ^33^ the lack of mature T cells may affect results, as the role of T lymphocytes in wound healing has recently begun to be elucidated.^34^, ^35^ Finally, suture technique (eg, simple interrupted vs running suture) affects wound tensile strength in murine models and should be carefully considered in study designs.^36^ In conclusion, in murine cancer models, neoadjuvant administration of NIR‐PIT shows no evidence of adverse effects on wound healing. These results support the premise that NIR‐PIT is likely to be a safe adjunct to surgical treatment of solid tumors, and future investigations of NIR‐PIT in the perioperative setting are merited.

## CONFLICTS OF INTEREST

The authors have no conflicts of interest to be reported.

## AUTHORS' CONTRIBUTION

AR, FI, TK, and RO mainly designed and conducted experiments, performed analysis and wrote the manuscript; HW, and AF performed analysis; PLC wrote and edited the manuscript and supervised the project; and HK planned and initiated the project, designed and conducted experiments, wrote and edited the manuscript, and supervised the entire project.

## Data Availability

The data that support the findings of this study are available from the corresponding author upon reasonable request.
